# Nectin expression in pancreatic adenocarcinoma: nectin-3 is associated with a poor prognosis

**DOI:** 10.1007/s00595-015-1126-2

**Published:** 2015-02-19

**Authors:** Hideki Izumi, Kenichi Hirabayashi, Naoya Nakamura, Toshio Nakagohri

**Affiliations:** 1 Department of Surgery, Tokai University School of Medicine, Kanagawa, Japan; 2 Department of Pathology, Tokai University School of Medicine, 143 Shimokasuya, Isehara, Kanagawa, 259-1193 Japan

**Keywords:** Pancreatic adenocarcinoma, Nectins, E-cadherin, Cell adhesion molecules, Prognosis

## Abstract

**Purpose:**

Nectins are cell adhesion molecules that regulate the formation of adherens junctions and are linked with E-cadherin-based cell–cell adherens junctions. In pancreatic cancer, the expression of E-cadherin and nectins is considered to be related to metastasis, invasion and prognosis.

**Methods:**

We evaluated the distribution of cells that were positive for nectin subtypes and E-cadherin using immunohistochemistry in specimens of human pancreatic adenocarcinoma, and correlated these results with the clinicopathological features and patient outcomes.

**Results:**

The immunohistochemical distribution of nectin-1 and E-cadherin showed a good correlation (*r* = 0.523, *p* < 0.01). Tumors over 4 cm in diameter had more intense staining for nectin-4 than smaller tumors (*p* = 0.035). Nectin-2 expression correlated with a poorer histological grade (*p* = 0.04). The cases that showed diffuse nectin-3 expression had a better prognosis than those with negative expression (*p* = 0.018).

**Conclusion:**

Our results showed that the expression of nectin-3 in pancreatic cancer can be a prognostic factor.

## Introduction

Pancreatic carcinoma is a lethal disease, and the incidence rates are almost equal to the mortality rates. A major factor that contributes to the high mortality rate is the propensity of pancreatic cancer cells to invade local tissues and disseminate widely throughout the body. The disruption of cell–cell adhesion is one of the important factors that results in the invasion and metastasis of carcinoma cells. Therefore, it would be indispensable to clarify the molecular mechanisms underlying cell adhesion to better understand the invasion and metastasis of cancer cells.

The epithelial-mesenchymal transition (EMT) is a phenomenon where the epithelial features of cells change to mesenchymal features, which can induce the loss of their cell polarity; this is associated with invasion and metastasis [1, 2]. E-cadherin, one of the factors associated with the EMT, is a member of the transmembrane glycoprotein family that is responsible for epithelial cell–cell adhesion, and the molecule is expressed by epithelial cells [3]. A reduction or loss of E-cadherin expression in cancer cells results in the destruction of the junctional structure, which can affect intercellular adhesion, which promotes tumor migration and metastasis [4, 5]. The loss of E-cadherin expression is related to the survival and prognosis of a number of cancers, such as extrahepatic bile duct carcinoma, pulmonary adenocarcinoma and pancreatic carcinoma [6].

Nectins are Ca^+^-independent [2] immunoglobulin-like cell adhesion molecules that regulate the formation of adherens junctions and tight junctions by epithelial cells [9]. They also participate in regulating cellular activities such as cell polarization and migration [10, 11]. The cytoplasmic region of nectins binds afadin, which directly connects to the actin cytoskeleton. The nectin–afadin complex is recruited to the E-cadherin-based cell–cell adherens junctions [10, 12]. Four subtypes of nectins are known: nectin-1, nectin-2, nectin-3 and nectin-4. Nectins-1, -2 and -3 are ubiquitously expressed in a variety of cells, whereas nectin-4 is mainly restricted to the placenta in humans [11, 13]. Recently, the expression patterns and characteristics of nectins in various tumors have been reported. For example, uterine cervical squamous cell carcinomas expressed nectin-1, but the advancing edge of the tumor nests of this tumor type had absent or reduced nectin-1 expression compared with the center of the tumor nests [14]. High nectin-2 expression in gallbladder adenocarcinoma and squamous cell aden squamous carcinoma has been shown to be related to a poor prognosis [15]. In non-small-cell lung cancer, a high level of nectin-4 expression is associated with a poor prognosis [16], and the serum level of nectin-4 was significantly higher in non-small-cell lung cancer patients than healthy tissue donors [16]. Serum nectin-4 has been identified as a marker of disease progression and therapeutic efficiency, and has been correlated with the number of metastases in breast carcinoma [17]. Similarly, the suppression of nectin-1 reduced the levels of cytokeratins-18, beta-catenin, claudine-3 and E-cadherin expression in breast cancer cell lines [18]. The membranous nectin-3 expression has been reported to be related to a poor prognosis in lung adenocarcinoma patients [19]. Nuclear factor *κ*B activity is associated with the expression of its downstream target genes, which leads to tumor progression, and was associated with the prognosis in an experimental model of pancreatic cancer [20]. However, to the best of our knowledge, there have been no evaluations of the importance of the expression of different nectin subtypes in pancreatic adenocarcinoma with regard to the clinicopathological features of patients. However, the expression of E-cadherin and nectins is considered to be related to the metastasis, invasion and prognosis of pancreatic cancer.

In the present study, we performed immunohistochemical staining for E-cadherin and the nectin subtypes in 49 pancreatic adenocarcinomas to elucidate whether the expression of E-cadherin and nectins was correlated with the clinicopathological features and clinical outcomes of the patients. In addition, we evaluated the correlation between the E-cadherin and nectin expression in pancreatic adenocarcinomas.

## Materials and methods

### Cases and sample preparation

A total of 49 IDC patients were examined in the present study. We only evaluated the cases of invasive adenocarcinoma, but eliminated adenocarcinomas derived from intraductal papillary mucinous neoplasms or special histological tumor types, such as aden squamous carcinoma or anaplastic carcinoma. The patients in our study had undergone surgical resection from 2000 to 2005 at Tokai University Hospital; all had undergone a routine histological diagnosis. The histological materials were fixed in formalin and embedded in paraffin; the sections were cut into 4 μm-thick sections and subjected to hematoxylin–eosin (HE) staining.

### Immunohistochemistry

Immunohistochemical staining for nectin-1, -2, -3, -4 and E-cadherin was performed. The formalin-fixed and paraffin-embedded materials were cut into 4 μm-thick sections, which were deparaffinized in xylene and rehydrated through a graded ethanol series of decreasing concentrations to distilled water. The immunohistochemical staining for nectin-3 (polyclonal; dilution, 1:100; Santa Cruz Biotechnology, CA, USA) and E-cadherin (clone 36 B5; dilution, 1:20; Leica Microsystems, Newcastle upon Tyne, United Kingdom) was performed using the Dako autostainer (Dako, Glostrup, Denmark) with antigen retrieval by autoclaving the samples for 15 min in a sodium citrate buffer at pH 6.0. The antibodies were detected using EnVision Plus (Dako) with 3,3′-diaminobenzidine as the chromogen. The immunohistochemical staining for nectin-1 (anti-PVRL1; polyclonal; dilution, 1:100; Sigma, MO, USA), nectin-2 (anti-PVRL2; polyclonal; dilution, 1:100; Sigma) and nectin-4 (anti-PVRL4; polyclonal; dilution, 1:100; Sigma) was completed using the BondMax (Leica) reagents according to the manufacturer’s manual. Antigen retrieval was performed by treatment with ER2 (Leica) for 30 min for nectin-1 and nectin-2, and with ER1 (Leica) for 30 min for nectin-4. Appropriate positive and negative tissue control samples were used.

### Evaluation of the immunohistochemical staining

The percentage of tumor cells that expressed nectins or E-cadherin in the cytoplasm or the membrane in the total number of tumor cells was scored as follows: less than 5 % positive cells, score 0 (negative); 5–29 %, score 1; 30–59 %, score 2 and over 60 %, score 3. The immunohistochemical results were evaluated by H.I. and K.H.

### Statistical analysis

All statistical analyses were performed using the SPSS version 19 software program (IBM Japan, Tokyo, Japan). For the statistical analysis, the Mann–Whitney *U* test and Kruskal–Wallis test were used to evaluate the differences between the immunohistochemical scores of nectins or E-cadherin and the clinicopathological findings. Spearman’s rank correlation coefficient was used to correlate the immunohistochemistry scores between the nectins and E-cadherin. Life-table probabilities for the overall survival were calculated using the Kaplan–Meier method, and the differences in survival between the subgroups were compared with the log-rank test. To define independent risk factors for the prognosis, a multivariate analysis was performed using a Cox proportional hazards model. A value of *p* < 0.05 was considered to be significant.

## Results

### Clinical features

The mean age of the 49 patients with pancreatic adenocarcinoma was 67 years old (range 50–87). There were 24 males (49 %) and 25 females (51 %). The mean tumor size was 3.5 cm (range 1.5–6.0) in diameter. Eleven cases were dominated by well-differentiated adenocarcinoma (G1) (22 %), 36 were dominated by moderately differentiated adenocarcinoma (G2) (74 %) and two were dominated by poorly differentiated adenocarcinoma (G3) (4 %). There were 32 cases with lymph node metastasis (65 %) and 17 without lymph node metastasis (35 %). The mean follow-up period was 816 days (range 73–3,515). Forty-two out of the 49 patients died of the disease, whereas seven were still alive at the most recent follow-up. The clinical features have been summarized in Table 1.

**Table 1:** Clinical features of the cases

		Nectin-1			Nectin-2			Nectin-3			Nectin-4			E-cadherin		
		Mean (SD)	Median (range)	*p* value	Mean (SD)	Median (range)	*p* value	Mean (SD)	Median (range)	*p* value	Mean (SD)	Median (range)	*p* value	Mean (SD)	Median (range)	*p* value
Total number of cases, no.	49															
Mean age, years (SD)	67 (9.0)															
Sex, no. (%)																
Male	24 (49)	2.83 (0.098)	3.0 (1–3)	0.581	0.83 (0.098)	1.0 (0–2)	0.278	2.88 (0.069)	3.0 (2–3)	0.729	0.54 (0.134)	0 (0–2)	0.441	2.75 (0.109)	3.0 (1–3)	0.886
Female	25 (51)	2.92 (0.055)	3.0 (2–3)		0.68 (0.125)	1.0 (0–2)		2.84 (0.075)	3.0 (2–3)		0.8 (0.191)	1.0 (0–3)		2.8 (0.082)	3.0 (2–3)	
Mean size of tumor, (cm) (SD)	3.5 (1.1)															
<4.0 cm, no. (%)	33 (67)	2.85 (0.077)	3.0 (1–3)	0.516	0.79 (0.095)	1.0 (0–2)	0.529	2.85 (0.063)	3.0 (2–3)	0.806	0.48 (0.116)	0 (0–2)	0.035	2.73 (0.09)	3.0 (1–3)	0.330
>= 4.0 cm, no. (%)	16 (33)	2.94 (0.063)	3.0 (2–3)		0.69 (0.151)	1.0 (0–2)		2.88 (0.085)	3.0 (2–3)		1.06 (0.249)	1.0 (0–3)		2.88 (0.085)	3.0 (2–3)	
Histological grade, no. (%)																
Grade 1	11 (22)	3.0	3.0 (3)	0.374	0.45 (0.157)	0 (0−1)		2.91 (0.091)	3.0 (2−3)	0.695	0.91 (0.368)	0 (0–3)	0.935	2.91 (0.091)	3.0 (2−3)	0.398
Grade 2	36 (74)	2.83 (0.075)	3.0 (1–3)		0.81 (0.087)	1.0 (0−2)	0.040	2.83 (0.063)	3.0 (2−3)		0.61 (0.115)	0.5 (0–2)		2.72 (0.086)	3.0 (1−3)	
Grade 3	2 (4)	3.0	3.0 (3)		1.5 (0.5)	1.5 (1−2)		3.0	3.0 (3)		0.5 (0.5)	0.5 (0–1)		3.0	3.0 (3)	
Lymph node metastasis, no. (%)																
Positive	32 (65)	2.88 (0.074)	3.0 (1–3)	0.826	0.66 (0.106)	1.0 (0–2)	0.70	2.84 (0.065)	3.0 (2–3)	0.716	0.66 (0.153)	0 (0–3)	0.677	2.75 (0.090)	3.0 (1–3)	0.696
Negative	17 (35)	2.88 (0.081)	3.0 (2–3)		0.94 (0.104)	1.0 (0–2)		2.88 (0.081)	3.0 (2–3)		0.71 (0.187)	1 (0–2)		2.82 (0.095)	3.0 (2–3)	

*SD* standard deviation

### Nectin immunohistochemistry

**Fig. 1:**
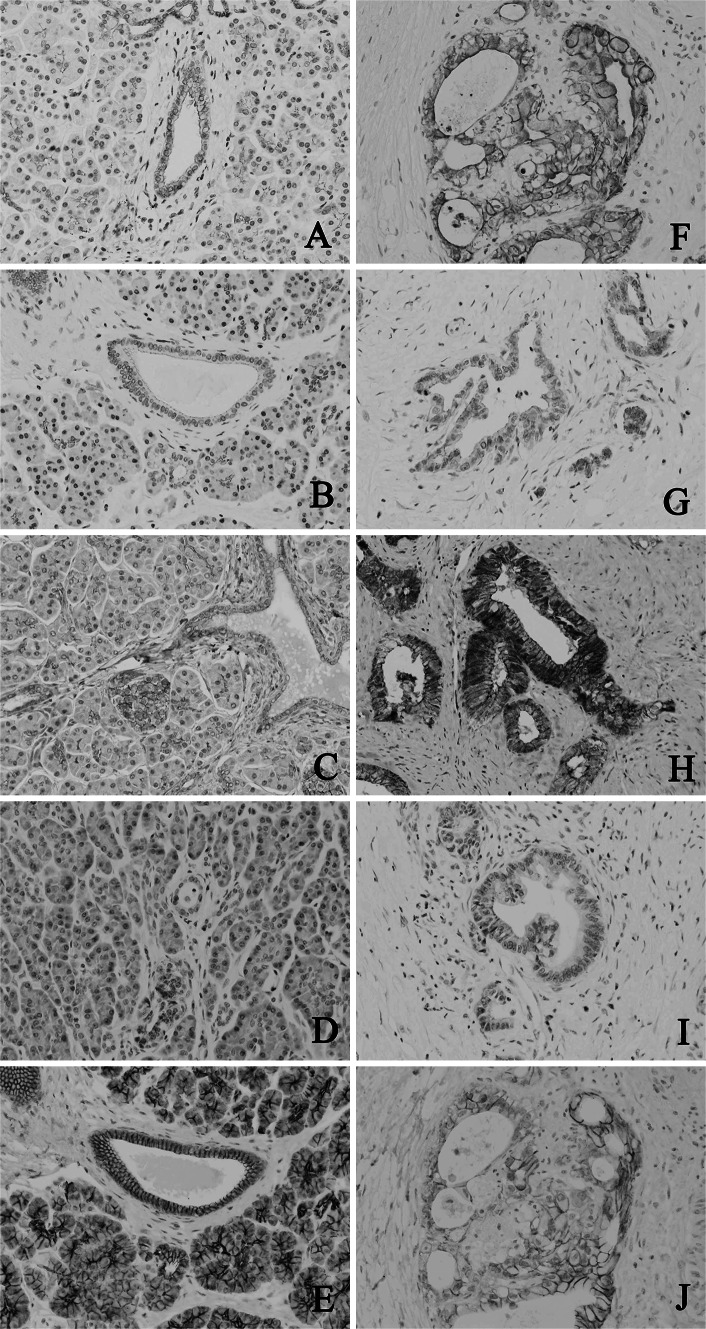
Representative immunohistochemical staining for nectin-1, nectin-2, nectin-3, nectin-4 and E-cadherin expression in the normal pancreatic parenchyma (**a** nectin-1, **b** nectin-2, **c** nectin-3, **d** nectin-4, **e** E-cadherin) and in adenocarcinoma (**f** nectin-1, **g** nectin-2, **h** nectin-3, **i** nectin-4, **j** E-cadherin)

Nectin-1 and nectin-3 showed both membranous and cytoplasmic expression in the intercalated ducts, intralobular ducts and interlobular ducts of the normal pancreas. Nectin-2 showed weak expression in the apical membrane in the intercalated ducts, intralobular ducts and interlobular ducts of the normal pancreas. There was no nectin-4 staining in the normal pancreatic ducts, including the intercalated ducts. The islet cells were negative for nectin-1, whereas nectin-2 and -3 showed membranous and cytoplasmic expression in the islet cells; however, the nectin-2 expression was weak. There was cytoplasmic expression of nectin-4 in the islet cells. In the adenocarcinoma cells, nectin-1 and -3 demonstrated both membranous and cytoplasmic expression. Nectin-2 and nectin-4 were also expressed in the membranes and cytoplasm of the adenocarcinoma cells, although the degree of expression was usually weak. Representative images showing the expression of nectin-1, -2, -3 and -4 in the normal pancreatic parenchyma and in pancreatic adenocarcinoma are presented in Fig. 1. The immunohistochemical scores for nectins and E-cadherin have been summarized in Table 2.

**Table 2:** Score of nectins and E-cadherin

	Score 0 (%)	Score 1 (%)	Score 2 (%)	Score 3 (%)	Mean score (SD)	Median
Nectin-1	0	1 (2)	4 (8)	44 (90)	2.88 (0.389)	3.0
Nectin-2	15 (31)	31 (63)	3 (6)	0	0.76 (0.56)	1.0
Nectin-3	0	0	7 (14)	42 (86)	2.86 (0.354)	3.0
Nectin-4	25 (51)	17 (35)	5 (10)	2 (4)	0.67 (0.826)	0
E-cadherin	0	1 (2)	9 (18)	39 (80)	2.78 (0.468)	3.0

*SD* standard deviation

Most of the pancreatic adenocarcinoma cases demonstrated diffuse nectin-1 (median score: 3.0, mean score: 2.88) and nectin-3 (median score 3.0, mean score: 2.86) expression. There were no cases with a score 0 for nectin-1 or nectin-3. In contrast, most of the pancreatic adenocarcinoma cases showed negative or focal staining for nectin-2 (median score: 1.0, mean score: 0.76) and nectin-4 (median score: 0, mean score: 0.67). A score of 3 for nectin-4 was observed in two cases (4 %), but there were no case with a score of 3 for nectin-2. There were no significant differences between the immunohistochemical scores of each nectin and the clinicopathological features, including lymph node metastasis and gender (Table 1). However, the tumors that were over 4 cm in diameter showed higher scores for nectin-4 than those that were less than 4 cm in diameter (*p* = 0.035) (Table 1). The nectin-2 expression correlated with the tumor grade (*p* = 0.04) (Table 1).

### E-cadherin immunohistochemistry

E-cadherin showed both membranous and cytoplasmic expression in the intercalated ducts, intralobular ducts and interlobular ducts of the normal pancreas and adenocarcinoma cells (Fig. 1e, i). E-cadherin was diffusely expressed in pancreatic adenocarcinoma (median score: 3.0, mean score: 2.78) (Table 2). There were no cases of pancreatic adenocarcinoma with a score of 0 for E-cadherin. There were no significant differences between the immunohistochemical scores for E-cadherin with regard to the clinicopathological features, including lymph node metastasis, the size of the tumor, patient gender or histological grade of the tumor (Table 1).

### Correlation of the immunohistochemical findings among nectins and E-cadherin

The immunohistochemical scores for nectin-1 and E-cadherin showed a good correlation (*r* = 0.523, *p* < 0.01), whereas there was no significant correlation in the other combinations among the nectins and E-cadherin (Table 3).

**Table 3:** Correlation of immunohistochemical score among nectins and E-cadherin

	Nectin-1	Nectin-2	Nectin-3	Nectin-4	E-cadherin
Nectin-1	1				
Nectin-2	0.224	1			
Nectin-3	0.071	0.131	1		
Nectin-4	−0.02	−0.217	−0.095	1	
E-cadherin	0.523*	0.242	0.247	0.051	1

* *p* < 0.01

### The relationship between nectins/E-cadherin expression and patient survival

**Fig. 2:**
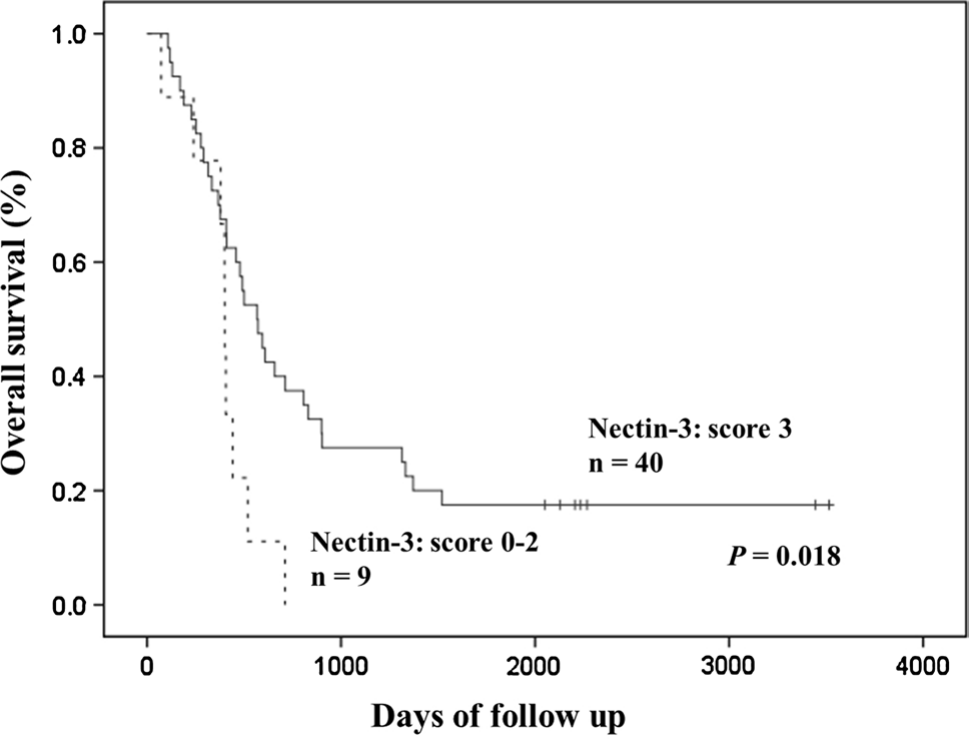
The overall survival curves according to the nectin-3 expression

There was no significant difference in the overall survival associated with the immunohistochemical scores for nectin-1, nectin-2, nectin-4 or E-cadherin. The patients with a score of 3 for nectin-3 showed a better prognosis than the cases with scores of 0–2 (*p* = 0.018) (Fig. 2). We performed a multivariate analysis that included the histological grade, lymph node metastasis and tumor size to assess the importance of the score of nectin-3. In the multivariate analysis, the score of nectin-3 (*p* = 0.015; hazard ratio 0.360; 95 % confidence interval 0.158–0.832) and tumor size (*p* = 0.014; hazard ratio 0.429; 95 % confidence interval 0.219–0.841) emerged as independent prognostic factors (Table 4).

**Table 4:** Multivariate analysis

Variables	Hazard ratio	95 % CI	*p* value
Grade (G1/G2, G3)	1.126	0.513–2.469	0.768
Lymph node metastasis	1.127	0.576–2.205	0.726
Tumor size (<4.0 cm/≧4.0 cm)	0.429	0.219–0.841	0.014
Score for nectin-3 (3/2–0)	0.360	0.158–0.832	0.015

*CI* confidence interval

## Discussion

The EMT plays a key role in the metastasis and invasion of various carcinomas. The disruption of the cell–cell adhesion of tumor epithelial cells, resulting in the EMT, causes tumor progression and is related to a poor prognosis of cancer patients. The EMT leads to the disruption of the adherens junctions that are composed of E-cadherin. In general, E-cadherin expression is strong in well-differentiated carcinomas, which often maintain their cell–cell adhesion and are less invasive; however, E-cadherin expression is reduced in undifferentiated cancers, which have lost their cell–cell adhesion and have strong invasive and metastatic tendencies [21]. E-cadherin expression was found to be absent or at low levels in the metastases of various tumors, including pancreatic cancer [8, 22]. Among pancreatic cancers, Guo et al. [22] reported that E-cadherin expression was significantly lower in pancreatic adenocarcinomas with lymph node metastasis compared with the adenocarcinomas without lymph node metastasis. Furthermore, several groups have reported that pancreatic cancers with the loss of E-cadherin had a poorer prognosis; therefore, E-cadherin expression has emerged as an independent prognostic factor [8, 26]. However, our data did not show any correlation of the E-cadherin expression with clinicopathological features such as lymph node metastasis, the tumor histological grade or prognosis. The reason why no significant difference was obtained was likely due to the small number of cases. Significant differences may appear if a larger number of cases were analyzed.

Nectins may be considered EMT-related molecules because they are transferred to E-cadherin via afadin and the actin cytoskeleton [10, 12]. There have been only a few studies about nectins related to the EMT and E-cadherin. Vetter et al. [18] reported that the suppression of nectin-1 reduced the E-cadherin expression in a breast cancer cell line which indicated that nectin-1 correlated with E-cadherin expression and the EMT. Our present results revealed that nectin-1 showed a good correlation with E-cadherin expression, whereas nectins-2, -3 and -4 showed a poor correlation with E-cadherin. These results indicated that nectin-1 is associated with the EMT via its correlation with E-cadherin. However, Matsushima et al. [27] reported that the expression of nectin-1α, one of the splice variants of nectin-1, was decreased in well-differentiated squamous cell carcinoma and basal cell carcinoma of the skin compared with that in the normal epidermis and Bowen’s disease, in spite of the preserved E-cadherin expression. In addition, Yu et al. [28] reported that the nectin-1 expression was inversely correlated with E-cadherin expression in head and neck squamous carcinoma cell lines. Therefore, the role of nectin-1 in tumors is still controversial, and will require further studies.

The expression of nectin-2 and nectin-4 has been reported to be associated with a poor prognosis in gallbladder carcinoma [15] and non-small-cell lung carcinoma [16], respectively. Although our present results showed no correlation between the expression of nectin-2 and nectin-4 and the patient survival, a higher score for nectin-2 was related to a poorer histological grade, and a higher score for nectin-4 was related to a larger tumor size. These results indicated that nectin-2 and nectin-4 may be associated with the extent of tumor malignancy in cases of pancreatic carcinoma. Furthermore, these findings suggest that the downregulation of nectin-4 is associated with the loss of cell-to-cell adhesion in pancreatic carcinoma. Further studies will be needed to elucidate the mechanism(s) by which pancreatic carcinomas downregulate nectin-4 expression, which can lead to the invasion and spread of such carcinomas.

Our present study showed that diffuse nectin-3 expression was associated with a good prognosis in pancreatic adenocarcinoma. However, Maniwa et al. [19] reported that membranous nectin-3 expression was an independent prognostic factor for lung adenocarcinoma Furthermore; they revealed that membranous nectin-3 expression correlated with a higher incidence of pleural invasion, pT factors, distant metastasis and vascular invasion. Our results demonstrated that a lack of nectin-3 expression and the tumor size were independent prognostic factors. The role of nectins in the extent of tumor malignancy, such as its invasion, metastasis and prognosis, may be different depending on the histological types and tumor origins.

In conclusion, the present study is the first to compare the expression of nectin subtypes and E-cadherin, and to correlate these with the clinicopathological features of pancreatic adenocarcinoma. Our results revealed that diffuse nectin-3 expression was associated with a good prognosis, a higher score for nectin-2 was related to a poorer histological grade and a higher score for nectin-4 was related to a larger tumor size. The malignant characteristics of pancreatic cancer cannot be explained by a single factor, and some factors are likely to participate in complicated ways that involve multiple pathways. In addition, it is thought that the expression of nectin-2 and nectin-4 in pancreatic cancer was not associated with the outcome; however, a significant difference was found in the pathological factor. Therefore, the roles of nectins in the invasion and metastasis of carcinoma cells have not yet been clarified.
